# Changes and socioeconomic factors associated with attitudes towards domestic violence among Vietnamese women aged 15–49: findings from the Multiple Indicator Cluster Surveys, 2006–2011

**DOI:** 10.3402/gha.v9.29577

**Published:** 2016-02-29

**Authors:** Oanh Thi Hoang Trinh, Juhwan Oh, Sugy Choi, Kien Gia To, Dung Van Do

**Affiliations:** 1 Faculty of Public Health, University of Medicine and Pharmacy, Ho Chi Minh City, Vietnam; 2 JW LEE Center for Global Medicine, Seoul National University College of Medicine, Seoul, Republic of Korea

**Keywords:** socioeconomic factors, intimate partner violence, attitudes, prevalence, Vietnamese

## Abstract

**Background:**

Understanding factors associated with domestic violence-supportive attitudes among Vietnamese women is important for designing effective policies to prevent this behavior. Previous studies have largely overlooked risk factors associated with domestic violence-supportive attitudes by women in Vietnam.

**Objective:**

This paper explores and identifies socioeconomic factors that contribute to domestic violence–supportive attitudes among Vietnamese women using data from the Multiple Indicator Cluster Surveys (MICS).

**Design:**

Secondary data from two cross-sectional studies (MICS 3, 2006, and MICS 4, 2011) with representative samples (9,471 and 11,663 women, respectively) in Vietnam were analyzed. The prevalence of supportive attitudes toward domestic violence and associations with age, residence region, area, education level, household wealth index, ethnicity, and marital status were estimated using descriptive statistics and multivariate Poisson models, giving estimates of relative risk.

**Results:**

Overall, the prevalence of acceptance of domestic violence declined between 2006 and 2011 in Vietnam (65.1% vs. 36.1%). Socioeconomic factors associated with women's condoning of domestic violence were age, wealth, education level, and living area. In particular, younger age and low educational attainment were key factors associated with violence-supportive attitudes, and these associations have become stronger over time.

**Conclusion:**

Higher educational attainment in women is an important predictor of women's attitudes toward domestic violence. To date, *Doi Moi* and the Vietnamese government's commitment to the Millennium Development Goals may have positively contributed to lowering the acceptance of domestic violence. Tailored interventions that focus on education will be important in further changing attitudes toward domestic violence.

## Background

According to the World Health Organization (WHO), domestic violence is a leading public health concern throughout the world. The prevalence is high and rising, and there are serious physical and mental health, as well as social, consequences (1, 2). Globally, 35% of women have indicated that they have suffered from physical or sexual violence, most of which can be described as intimate partner violence (2). A systematic review revealed a high prevalence of domestic violence rates among Asian households, with 41–61% of respondents suffering from intimate, physical, and/or sexual violence at some time during their lifetime (3).

Previous studies have drawn attention to the complexities of understanding Vietnamese women's attitudes toward domestic violence. Several studies have reported that beliefs and attitudes constituting gender equality have had a significant impact on attitudes (4, 5). Many studies have explored the male perpetrators’ perceptions of partner violence as well as social conceptions imposed upon the perpetrators. For example, a report from Ho Chi Minh City in 2006 revealed that 47 and 68% of men were identified as current and past perpetrators of intimate partner violence, respectively (6). These prevalence rates are higher than among Vietnamese-American immigrants and non-Vietnamese populations in the United States (4, 7). Influenced for centuries by patriarchal legal norms such as pro-male inheritance law, Vietnamese men have a higher position than women in their families and society, although this is not the case for those living outside Vietnam. This gender inequity, in part, explains women's economic dependency on their husbands, which may be a factor in their accepting habitual violence perpetrated by their husbands (8). Other demographic and socioeconomic factors in Vietnam, such as education, finances, age, marital status, and lifestyle, have been linked to abusive social norms toward women. Unfortunately this situation is far removed from a gender equality pathway (6).

Existing intervention programs have focused on preventing domestic violence either by altering micro-level factors such as attitudes and behaviors that contribute to violent responses, or through macro-level responses, such as altering economic, political, and cultural conditions (2). It has been noted that the previously formed attitudes of men contribute significantly to their committing violence against women, since men believe that such actions are acceptable (8). Research shows that economic hardship, traditional family values, beliefs in traditional female roles, and perceptions of racial discrimination can prevent Vietnamese-American women from accessing formal support systems when facing domestic abuse (8). It has been suggested that violence-prevention campaigns should focus on changing attitudes (2).

A qualitative study in Australia suggested that attitudes regarding domestic violence were associated with two clusters of factors – gender and culture – that are influential at multiple levels of society (8). Other reports in Australia (9) and the United States (10, 11) show that attitudes toward violence against women vary at individual, community, and social levels. Socioeconomic factors such as social disadvantage, economic dependency, Asian or African ethnicity, younger age, and social norms shape a person's attitudes toward domestic violence. A survey in Australia showed that age, gender, support for gender equality, and migration and settlement (Asian vs. European descent) were consistent predictors of violence-provoking attitudes, with measures of socioeconomic status less so (5).

Reports of three rounds of the global Multiple Indicator Cluster Surveys (MICS) in Vietnam from 2006 to 2013 (MICS3 in 2006, MICS4 in 2011, and MICS5 in 2013) show that the prevalence of supportive attitudes toward domestic violence among women declined considerably over time from 63.8 to 35.8 and 28.2% (12). Socioeconomic reforms in Vietnam may have helped (2, 8, 11), although this effect is not clear. Changes in attitudes and risk factors associated with domestic violence among Vietnamese women have not previously been reported. This study investigates associations between, and changes in, socioeconomic factors and domestic violence–supportive attitudes among Vietnamese women.

## Methods

### Data source

In this paper, secondary data from the MICS (2006 and 2011) were analyzed to show trends in domestic violence attitudes among Vietnamese women aged 15–49 cross-sectionally and over time (12). The General Statistics Office in collaboration with the Ministry of Health and the Ministry of Labour, Invalids and Social Affairs conducted the MICS. The United Nations Children's Fund (UNICEF) and the United Nations Population Fund provided financial and technical support for the MICS (12).

The MICS used different sampling designs in different years. In 2006, eight regions were included: the Red River Delta, the North West, the North East, the North Central Coast, the South Central Coast, the Central Highlands, the South East, and the Mekong River Delta. The regions were identified as the main sampling domains and the sample was selected in two stages. During the first stage, 250 census enumeration areas (EAs) were selected, and within each region 30–33 EAs were selected. A systematic sample comprising a third of households in each EA was drawn.

In 2011, six regions were included: the Red River Delta, the Northern Midland and Mountain area, the North Central and Central Coastal areas, the Central Highlands, the South East, and the Mekong River Delta. The urban and rural areas within each region were identified as the main sampling strata, and the sample was selected in two stages. Within each stratum, a specified number of census EAs were selected using the probability proportional to size method. A systematic sample of 20 households was drawn in each EA sample. Samples were stratified by region, urban, and rural areas.

In each of the survey years, the sample was stratified by regions and areas (rural and urban). Sampling weights, based on the Population and Housing Census 1999 and the Population Census 2009, were applied to give representative data for the regions in Vietnam (12). A total of 9,470 and 11,663 women aged 15–49 years were selected in the MICS in 2006 and 2011, respectively. Details of these surveys are published elsewhere (12).

### Domestic violence variable

The dependent variable *domestic violence–supportive attitudes* was binary. This was derived from a set of five questions as follows: Is a husband justified in hitting or beating his wife in the following situations: 1) if she goes out without telling him, 2) if she neglects the children, 3) if she argues with him, 4) if she refuses to have sex with him, and 5) if she burns the food? If women agreed with one or more of these five questions, they were identified as having domestic violence–supportive attitudes (12).

### Socioeconomic status variables

Sociodemographic variables included were as follows: age group (15–19, 20–29, 30–39, and 40–49), education level (illiterate, primary school, lower secondary school, upper secondary school, and tertiary education), location of residence (ecological area), area (urban, rural), ethnicity of head of household (Kinh or non-Kinh), marital status (married, formerly married, or single in union), and a household wealth index. The wealth index was based on household assets as a measure of economic status. A listing was constructed using methods recommended by the World Bank Poverty Network and UNICEF and described by Filmer and Pritchett (13). The household wealth index was computed by grouping households into tertiles from the poorest to the richest.

### Statistical analysis

Data were weighted and adjusted for stratification. Weights were calculated based on the population distribution of women aged 15–49 years in Vietnam (from the Population and Housing Census 1999 and the Population Census 2009) (12) to reduce sampling design bias. All analyses were performed using Stata/SE software version 12.0, with the *svyset* commands used to weight the data.

The distribution of characteristics in the study sample is presented by age group, region (ecological regions of Vietnam), area (urban, rural), education level, and wealth index tertiles in 2006 and 2011, using frequencies and percentages. The prevalence of supportive attitudes toward domestic violence is reported by sample characteristics. Proportions with 95% confidence intervals (CIs) are given.

The Pearson chi-square test was performed to test association between sociodemographic and domestic violence–supportive attitudes. Tests for linear trends are shown by ordinal categories, which indicate dose–response relationships. Prevalence ratios (PRs), representing the ratios of prevalence proportions, are estimated. Univariate and multivariate Poisson regressions were used to show PRs between explanatory variables and attitudes toward domestic violence. Crude and adjusted PRs were estimated showing similar results. Only the adjusted PRs with 95% CIs are reported here. The Wald test was performed (*p*<0.05) to find a best-fit model. Because time was significantly associated with the dependent variable, a time variable and interaction terms (time×age group and time×education) were included.

## Results

### Sample characteristics

There were 9,471 and 11,663 women interviewed in the MICS during 2006 and 2011, respectively (Table 1). There were higher proportions of respondents who lived in urban areas (31.5% vs. 25.1%), had completed lower secondary school (38.7% vs. 25.5%), and were married (71.5% vs. 65.7%) in 2011 compared with 2006. The distribution of age, region, area, education, marital status, wealth index, and household head's ethnicity differed significantly between 2006 and 2011 (*p*<0.001).

**Table 1:** Socioeconomic characteristics of the study sample of women aged 15–49, in the 2006 and 2011 MICS

	2006 (*n*=9,471)*			2011 (*n*=11,663)**		
	
Characteristics	*N*	%	% weighted	*N*	%	% weighted
Age group***						
15–19	1,851	19.5	18.8	1,769	15.2	14.6
19–29	2,587	27.3	26.7	3,418	29.3	29.3
30–39	2,491	26.3	26.5	3,379	29.0	29.8
40–49	2,542	26.8	28.0	3,097	26.6	26.3
Region***						
Red River Delta	1,335	14.1	21.5	1,682	14.4	20.3
Northern Midland and Mountain areas	2,128	22.5	15.5	1,970	16.9	16.3
Northern Central area and Central Coastal area	2,250	23.8	21.3	1,868	16.0	20.8
Central Highlands	1,169	12.3	3.7	2,078	17.8	5.8
South East	1,357	14.3	17.2	2,116	18.1	17.8
Mekong River Delta	1,232	13.0	20.8	1,949	16.7	19
Area***						
Urban	2,380	25.1	26.5	5,183	44.4	31.5
Rural	7,091	74.9	73.5	6,480	55.6	68.5
Education***						
None	1,327	14.5	12.4	612	5.2	4.1
Primary	1,021	10.8	10.9	1,883	16.1	16.3
Lower secondary school	2,424	25.6	25.5	4,244	36.4	38.7
Upper secondary school	3,210	33.9	35.0	2,830	24.3	24.3
Tertiary	1,444	13.3	16.2	2,094	18.0	16.6
Marital status***						
Married/in union	6,208	65.5	65.7	8,194	70.3	71.5
Formerly married/in union	367	3.9	3.9	510	4.4	4.1
Single/in union	2,896	30.6	30.4	2,959	25.4	24.4
Wealth index***						
Poorest	3,157	33.3	28.2	4,039	33.3	33.7
Middle	3,157	33.3	35.6	4,039	33.3	33.6
Richest	3,157	33.3	36.2	4,037	33.3	32.7
Ethnicity***						
Kinh/Hoa	7,302	77.1	84.8	9,836	84.3	86.1
Minorities	2,169	22.9	15.2	1,827	15.7	13.9

* Weighted data based on the Population and Housing Census 1999

** weighted data based on the Population Census 2009

*** chi-square test with *p*<0.001.

### Changes in attitudes toward domestic violence

**Fig. 1:**
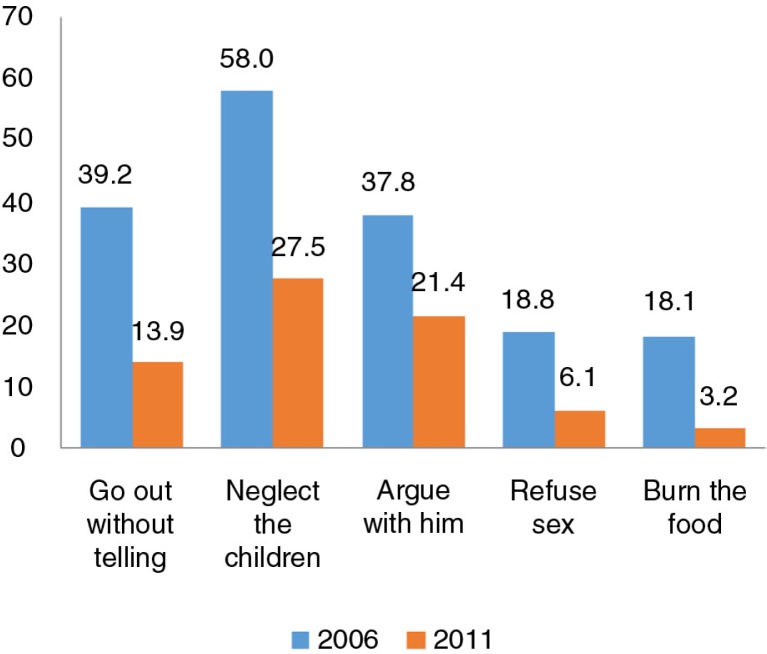
Prevalence of acceptance of domestic violence by women aged 15–49, 2006 and 2011.

**Fig. 2:**
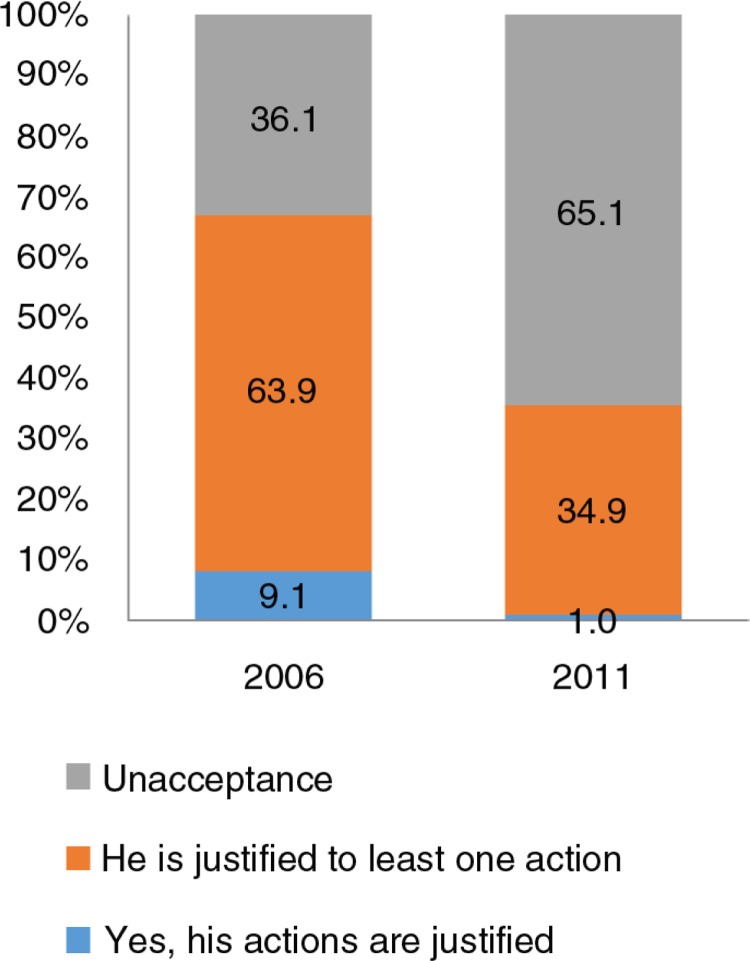
Change in attitudes toward domestic violence by women aged 15–49, 2006 and 2011.

Figure 1 shows the positive changes in attitudes toward domestic violence among Vietnamese women who answered five situation-based questions in 2006 and 2011. Overall there was a reduction in pro-violence attitudes for all of the questions – burning food, refusing sex, going out without telling, arguing with the husband, and neglecting the children (ordered from highest to lowest). Figure 2 also demonstrates a positive trend in attitudes toward domestic violence among Vietnamese women in 2011 compared with 2006 (65.1% vs. 36.1%, respectively). The prevalence of women who did not accept domestic violence increased by 29% between 2006 and 2011.

### Association between socioeconomic status and supportive attitudes toward domestic violence

In 2006, women living in rural areas had a higher risk of accepting domestic violence compared with those living in urban areas (PR=1.38, 95% CI: 11.13–1.67, *p*<0.01) (Table 2). The acceptance of domestic violence was more prevalent among the poor, less educated, those of Kinh descent, and those who were married. The results show that tertiary-level educational attainment (PR=0.86, 95% CI: 0.74–0.99, *p*<0.05), single marital status (PR=0.72, 95% CI: 0.63–0.82, *p*<0.001), non-Kinh ethnicity (PR=0.88, 95% CI: 0.82–0.95, *p*<0.01), and being in the richest or middle wealth tertile (PR=0.77, 95% CI: 0.70–0.85, *p*<0.001; and PR=0.93, 95% CI: 0.89–0.98, *p*<0.01, respectively) were protective. Chi-square tests for education (*p*<0.001) and wealth (*p*<0.001) indicate dose–response relationships (i.e. higher education was associated with less acceptance as was higher wealth).

**Table 2:** Multivariate Poisson model of association between socioeconomic status and attitudes of acceptance of domestic violence by women aged 15–49, 2006 and 2011

	2006 (*n*=8,830)		2011 (*n*=11,049)	
	
	Adjusted PR	95% CI	Adjusted PR	95% CI
Age group				
15–19	1.00	–	1.00	–
19–29	0.94	0.88–1.02	0.91	0.82–1.00*
30–39	0.98	0.90–1.08	0.84	0.77–0.93**
40–49	0.99	0.89–1.09	0.88	0.77–1.01
Region				
Red River Delta	1.00	–	1.00	–
Northern Midland and Mountain areas	0.96	0.87–1.07	1.31	1.17–1.47***
Northern Central area and Central Coastal area	1.18	0.97–1.43	1.43	1.36–1.52***
Central Highlands	0.94	0.86–1.04	1.12	1.03–1.22*
South East	0.74	0.55–0.98*	0.78	0.63–0.97*
Mekong River Delta	1.05	0.92–1.19	1.17	1.03–1.33*
Area				
Urban	1.00	–	1.00	–
Rural	1.38	1.13–1.67**	1.00	0.88–1.13
Education^a^				
None or primary school	1.00	–	1.00	–
Lower secondary school	1.07	0.99–1.15	0.89	0.83–0.96**
Upper secondary school	0.98	0.89–1.09	0.69	0.65–0.75***
Tertiary	0.86	0.74–0.99*	0.43	0.36–0.51***
Marital status				
Married/in union	1.00	–	1.00	–
Formerly married/in union	0.92	0.85–1.00	1.04	0.88–1.23
Single/in union	0.72	0.63–0.82***	0.93	0.83–1.04
Wealth index^a^				
Poorest	1.00	–	1.00	–
Middle	0.93	0.89–0.98**	0.97	0.89–1.04
Richest	0.77	0.70–0.85***	0.73	0.60–0.88**
Ethnicity				
Kinh	1.00	–	1.00	–
Non-Kinh	0.88	0.82–0.95**	1.02	0.91–1.14

* Wald test with *p*<0.05

** *p*<0.01

*** *p*<0.001

a linear test for trend across categories, *p*<0.01. PR, prevalence ratio; adjusted PR for all variables in the tables.

Women in the South East region had lower levels of acceptance regarding domestic violence in both survey years (PR=0.74, 95% CI: 0.55–0.98, *p*<0.05; and PR=0.78, 95% CI: 0.63–0.97, *p*<0.05, respectively). There was a concerning shift in the direction of association in several regions. For example, women living in the Northern Midland and Mountain areas and Central Highlands had lower levels of acceptance in 2006, but in 2011 they had more supportive attitudes toward domestic violence. Except for women living in the Red River Delta and South East areas, other women were at higher risk of accepting domestic violence in 2011 compared with 2006. In 2011, educational level was a strong predictor of women's supportive attitudes toward domestic violence, even at the lower secondary school level (*p*<0.01).

### Predictors of changes in attitudes toward domestic violence over time

**Fig. 3:**
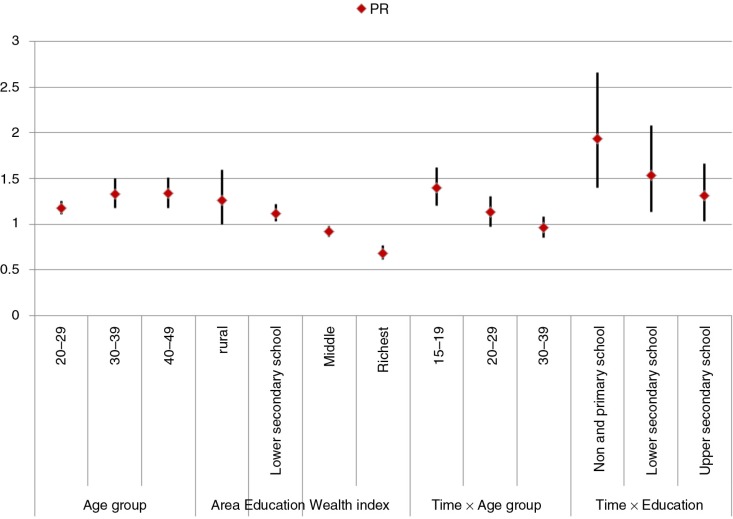
Trend in changing attitudes toward domestic violence by women aged 15–49, 2006 and 2011. PR, prevalence ratio; time×age group, interaction term between time and age group; time×education, interaction term between time and education level.

In the study, time was a significant effect modifier of the association between socioeconomic variables such as age, education, and acceptance of domestic violence among Vietnamese women. Figure 3 shows that women with low educational attainment and younger women were more likely to accept domestic violence between 2006 and 2011.

## Discussion

Vietnam recognizes domestic violence as an important issue related to human rights. Laws on domestic violence prevention and control were established in 2007 to prevent future violence (14, 15). Strengthening the law on domestic violence may have contributed to the decreasing trend in pro-violence attitudes among women in Vietnam between 2006 and 2011, Many Vietnamese are now aware that committing domestic violence is illegal. Other possible reasons include economic development and the aging population (16). However Vietnam still lags behind other countries (5).

In 2003, a study among men aged 17–75 years old in Ho Chi Minh City reported that 68% had been physical abusers and 47% were current physical abusers (6). In Vietnam, due to patriarchal ideology, women are supposed to obey their husbands, and a sign of a ‘good wife’ is acceptance and cover-up of violent actions perpetrated by husbands (15).

Factors associated with attitudes toward domestic violence have been shown previously, but mainly as part of research undertaken in developed countries. These studies reported that gender (5, 8, 17, 18), age (5, 18, 19), educational level (18–20), wealth index (19, 21), support for gender equality (5, 19), migration and settlement factors (19, 20), rural residence (19, 20), and culture/acculturation (18) are associated with the acceptance of domestic violence. Similarly, our findings also suggest that age, wealth index, educational level, and living area are significant predictors for much-needed change in attitudes in Vietnam. Kim-goh and colleagues had similar findings in their study, which showed that gender, education, and acculturation level were significant predictors of attitudes toward domestic violence among Korean and Vietnamese populations (18).

Although factors associated with attitudes toward domestic violence in this study in Vietnam were similar to those in other countries, there are some differences in the direction and strength of association. For example, factors such as ethnicity and living regions were significantly associated with the acceptance of domestic violence by women in Vietnam, whereas to our knowledge this association has not been demonstrated in other countries. With influences such as gender equality movements, modernization, and globalization, the acceptance of domestic violence in Vietnam may decrease in the future (20, 22).

There are several limitations in this study. Due to the restricted number of variables available in the MICS, other factors such as gender (17, 18, 23), husbands’ attitudes (6, 7), access to information and autonomy in household decisions (20), father-to-mother violence (21), parents’ educational level (21), and the numbers of live children (21) have not been explored. Another concern is that the different sampling strategies used in the survey years may have underestimated the prevalence of violence-supportive attitudes in 2011 compared with 2006. However, both datasets were weighted, which means that they were representative samples.

Importantly, our results are informative because they demonstrate many common socioeconomic factors that influence attitudes, as well as some other predictors of attitude change. This study has partly closed the gap in knowledge about how various factors may impact the acceptance of domestic violence by Vietnamese women.

## Conclusion

Domestic violence became less acceptable in Vietnam from 2006 to 2011. This is a positive finding but there is still a long way to go. Education plays an important role. *Doi Moi* and the Vietnamese government's commitment to the Millennium Development Goals may have helped. In particular, tailored interventions that focus on education will be important in bringing about future change in attitudes.
